# The Fault Diagnosis of Rolling Bearings Is Conducted by Employing a Dual-Branch Convolutional Capsule Neural Network

**DOI:** 10.3390/s24113384

**Published:** 2024-05-24

**Authors:** Wanjie Lu, Jieyu Liu, Fanhao Lin

**Affiliations:** School of Mechanical Engineering, Liaoning Technical University, Fuxin 123000, China; liujieyu980203@163.com (J.L.); damahou534@163.com (F.L.)

**Keywords:** dual-branch, capsule neural network, fault diagnosis, rolling bearing

## Abstract

Currently, many fault diagnosis methods for rolling bearings based on deep learning are facing two main challenges. Firstly, the deep learning model exhibits poor diagnostic performance and limited generalization ability in the presence of noise signals and varying loads. Secondly, there is incomplete utilization of fault information and inadequate extraction of fault features, leading to the low diagnostic accuracy of the model. To address these problems, this paper proposes an improved dual-branch convolutional capsule neural network for rolling bearing fault diagnosis. This method converts the collected bearing vibration signals into grayscale images to construct a grayscale image dataset. By fully considering the types of bearing faults and damage diameters, the data are labeled using a dual-label format. A multi-scale convolution module is introduced to extract features from the data and maximize feature information extraction. Additionally, a coordinate attention mechanism is incorporated into this module to better extract useful channel features and enhance feature extraction capability. Based on adaptive fusion between fault type (damage diameter) features and labels, a dual-branch convolutional capsule neural network model for rolling bearing fault diagnosis is established. The model was experimentally validated using both Case Western Reserve University’s bearing dataset and self-made datasets. The experimental results demonstrate that the fault type branch of the model achieves an accuracy rate of 99.88%, while the damage diameter branch attains an accuracy rate of 99.72%. Both branches exhibit excellent classification performance and display robustness against noise interference and variable working conditions. In comparison with other algorithm models cited in the reference literature, the diagnostic capability of the model proposed in this study surpasses them. Furthermore, the generalization ability of the model is validated using a self-constructed laboratory dataset, yielding an average accuracy rate of 94.25% for both branches.

## 1. Introduction

Rolling bearings are pivotal components in rotating machinery and also susceptible to damage. Their operational state directly impacts equipment performance. Reaching an accurate diagnosis of faults in bearing vibration signals poses a formidable challenge due to factors such as diverse operating conditions and the presence of noise interference. Therefore, precise fault diagnosis of bearings plays a crucial role in ensuring equipment safety and stable operation.

Currently, the primary methods for diagnosing faults in rolling bearings include model-based diagnosis, signal analysis, and data-driven approaches. Model-based diagnostic techniques commonly used are residual analysis (RA), state estimation (SE), parameter identification (PI), etc. [1]. The signal analysis method determines whether there is a fault by analyzing system or equipment signals. This approach typically involves analyzing features such as frequency F and amplitude A of the signals to determine the type and location of faults [2]. Signal analysis mainly includes traditional signal analysis and diagnosis methods in the time domain [3], frequency domain [4,5] and time–frequency domain. The collected vibration signals are projected to the time domain to extract different parameters, such as dimensional constants such as peak value, mean value, and variance, and dimensionless parameters such as peak index, kurtosis, and skewness. Bearing fault diagnosis using data-driven approaches refers to predicting and diagnosing equipment faults through large-scale historical data analysis and modeling using techniques such as machine learning and data mining [6]. Data-driven methods for rolling bearing fault diagnosis can be further divided into machine learning-based methods and deep learning-based methods [7].

Machine learning-based fault diagnosis methods for rolling bearings involve feature extraction and model training using bearing fault data, enabling the classification and prediction of bearing faults. Common machine learning methods include logistic regression (LR) [8], random forest (RF) [9], and artificial neural networks (ANNs) [10]. Chen Yin et al. [11] proposed a novel approach based on improved ensemble noise-assisted empirical mode decomposition (IENEMD) and adaptive threshold denoising (ATD). This method addresses the mixing problem in the original EMD while reducing noise interference. Yongbo Li et al. [12] introduced a new method for rolling bearing fault diagnosis based on adaptive multiscale fuzzy entropy (AMFE) and support vector machines (SVM). Unlike existing fuzzy entropy algorithms such as MFE, AMFE adaptively determines scales using robust Hermite local mean decomposition (HLMD). Although these methods are capable of extracting and identifying desired fault features, they often rely heavily on complex mathematical tools to extract signal characteristics from bearing faults. Therefore, traditional diagnostic methods for extracting fault features excessively depend on knowledge related to signal processing.

The method for diagnosing rolling bearing faults, based on deep learning, involves constructing a deep neural network model to automatically extract features and predict classifications of bearing fault data. Common deep learning methods include convolutional neural networks (CNNs) [13] and recurrent neural networks (RNNs) [14]. Guo Liang [15] determined the effectiveness of feature extraction through correlation analysis and utilized the useful feature set as input for an RNN, achieving superior diagnostic performance compared to self-organizing map methods. However, in practical engineering applications, vibration signals collected by sensors are often influenced by environmental noise. Additionally, the rotational speed of rolling bearings is affected by varying load conditions. Therefore, when diagnosing bearing faults under the interference of noise and varying operating conditions, common deep learning neural networks exhibit limited accuracy and generalization ability. Capsule neural networks (CapsNets) [16,17], which differ from traditional neural networks as they consist of neurons composed of vectors, can effectively extract and store more detailed features from input data while minimizing information loss in feature representation. However, due to its relatively simple initial structure, CapsNet does not further extract original image data features, resulting in incomplete extraction of detailed features; thus, its feature extraction capability still requires improvement.

This paper proposes a dual-branch CapsNet model for diagnosing faults in rolling bearings based on the aforementioned problem analysis. This approach transforms one-dimensional time series into grayscale image inputs and utilizes the dual branches to independently detect both fault types and damage diameters. By doing so, it effectively reduces the feature quantity in each branch while maintaining accuracy, thereby alleviating the challenge of feature extraction. Consequently, this model enhances the accuracy of multi-state classification for rolling bearings and strengthens diagnostic capability for novel faults. Furthermore, it demonstrates robustness against noise interference and varying operating conditions.

## 2. The Foundational Theory

### 2.1. Preprocessing of Grayscale Images

**Figure 1:**
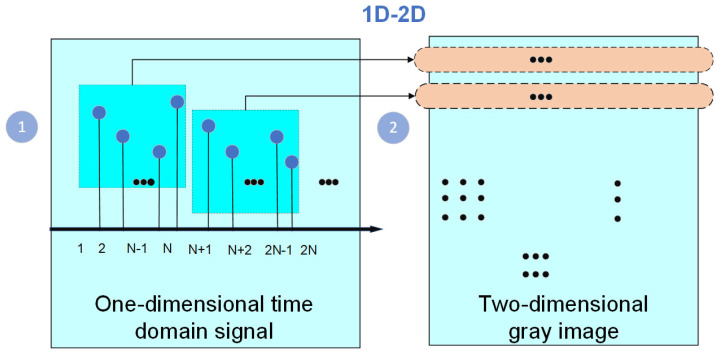
Diagrammatic representation of grayscale images.

In fault diagnosis methods driven by data, it is crucial to thoroughly investigate potential mapping relationships within the dataset. The process of transforming one-dimensional time series signals into grayscale images involves mapping the temporal sequence onto the horizontal axis and representing its values along with signal intensity on the vertical axis, resulting in a two-dimensional grayscale image. Grayscale values are adjusted based on signal intensity using either linear or nonlinear mappings. Figure 1 illustrates this principle, and specific conversion steps are outlined below.

1. Re-scale the one-dimensional temporal signal to ensure its values are normalized within the range of 0 and 1;
2. The normalized signal is sampled at fixed time intervals to obtain a discrete temporal signal;
3. Employ interpolation techniques to estimate the continuous temporal signal by leveraging the neighboring sampling points;
4. Each point on the continuous curve corresponds to a pixel point on the grayscale image. The value of each point on the curve is then converted to the grayscale value of the corresponding pixel by selecting the appropriate grayscale mapping method.

Furthermore, grayscale images offer two distinct advantages: Firstly, this method effectively preserves the temporal characteristics of the original signal to a maximum extent. Secondly, it simplifies the preprocessing work by eliminating the need for extracting new parameter indicators. This not only saves valuable preprocessing time but also reduces the burden on researchers in terms of fault information requirements.

### 2.2. Capsule Neural Network

**Figure 2:**
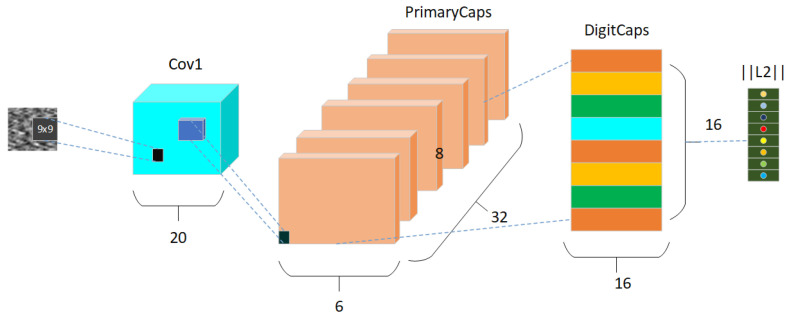
Capsule neural network structure.

The CapsNet [18,19] is a novel network model proposed by Geoffrey Hinton in October 2017. This architecture comprises an input layer, a convolutional layer, a primary capsule layer, a digit capsule layer, and a fully connected layer. Unlike conventional neural networks, the fundamental unit of the capsule neural network is represented by capsules that encapsulate local object features holistically. Each capsule consists of multiple neurons and both its input and output are vector representations. While the length of the vector signifies probability as seen in traditional neurons, its direction conveys additional information. A key highlight of this architecture lies in its utilization of a dynamic routing mechanism to replace the max pooling method employed in conventional convolutional neural networks. This approach effectively circumvents information loss caused by pooling layers and results in enhanced recognition accuracy. The model is shown in Figure 2.

The loss function of CapsNet consists primarily of two components: margin loss and reconstruction loss. Margin loss is employed to penalize incorrect identification outcomes in terms of both false negatives and false positives. It is computed using the following formula.

(1) $$L_{h}=T_{h}\cdot {\left(\max(0,m)-\left\|v_{h}\right\|\right)}^{2}+\lambda \cdot (1-T_{h})\cdot \max(0,\left\|v_{h}\right\|-m)$$

In the equation, *T_h_* represents the classification indicator function (1 if class h exists, 0 otherwise); *ν_h_* represents the output data of the network; *m*+ is the upper bound that penalizes false positives; and *m*− is the lower bound that penalizes false negatives. For this study, we have chosen empirical values of *m*+ = 0.9 and *m*− = 0.1. *λ* is a proportion coefficient that adjusts the weight between them with an initial default value of 0.5.

The reconstruction loss, also known as the mean squared error (MSE) loss, primarily focuses on the task of image reconstruction. Following the passage through the capsule layer, three fully connected layers are constructed to generate output values that precisely match those of the original data points. Subsequently, the squared sum of the distances between the original and output data is computed as a measure for evaluating this process. The total loss encompasses both margin loss and a multiplied reconstruction loss, where a multiplication factor *λ* ranging from 0.0001 to 0.001 is incrementally applied in this calculation in a stepwise manner. Consequently, margin loss is generally regarded as its principal indicator.

### 2.3. Inception Module

**Figure 3:**
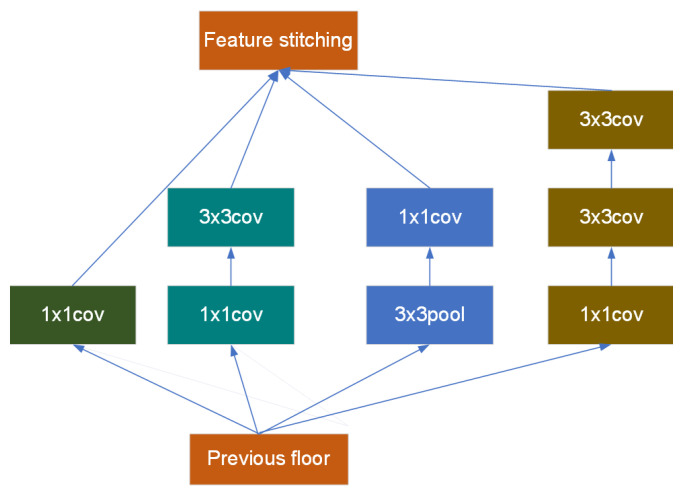
The InceptionV2 structure.

The Inception module was initially introduced in the deep learning architecture known as GoogleNet, wherein convolutional kernels of varying sizes are stacked together to enhance network width and extract comprehensive feature information. Moreover, it incorporates 1 × 1 scale convolutional kernels to reduce input feature map dimensionality, thereby decreasing parameters and accelerating network computation and training speed. By incorporating activation functions, the nonlinear expressive capability of multiple layers of convolutional kernels is enhanced while increasing the depth of convolutional layers helps prevent gradient vanishing. The structure of Inception V2 is depicted in Figure 3.

Inception V1 primarily employs multi-scale convolutional kernels and incorporates 1 × 1 convolutions to reduce the dimensionality of feature maps, effectively decreasing computational complexity. In V2, two smaller 3 × 3 convolutional kernels are utilized instead of a larger 5 × 5 kernel in V1. This approach ensures an expanded receptive field while reducing the number of parameters, thereby preventing expression bottlenecks and enhancing linear expressive capabilities.

### 2.4. The Attention Mechanism in Coordinate Automata (CA)

**Figure 4:**
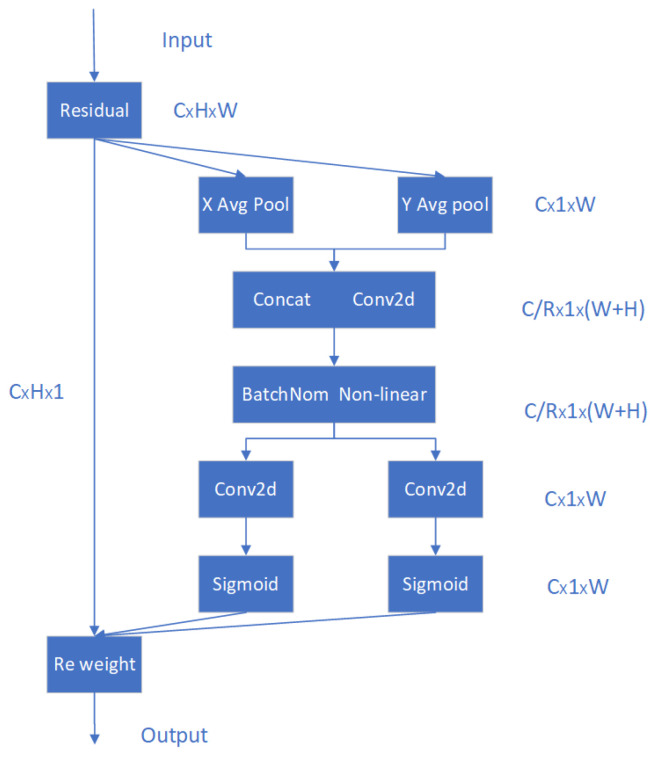
CA mechanism of attention.

The attention mechanisms commonly employed in constructing convolutional neural networks currently encompass the SE attention mechanism (which incorporates attention to the channel dimension), ECA attention mechanism (which applies channel-wise attention weighting), CBAM attention mechanism (composed of both channel and spatial attentions), and CA attention mechanism. When computing channel attentions for the first three mechanisms, global max pooling/average pooling is typically utilized, resulting in a loss of spatial information pertaining to objects. However, the CA mechanism integrates positional information into channel attentions, thereby circumventing this issue. The schematic diagram illustrating its operational principle is depicted in Figure 4.

The steps of the CA attention mechanism are as follows:

1. The input feature map is globally average pooled in both width and height directions to obtain feature maps in both dimensions;
2. The two parallel stages are merged by transposing the width and height onto the same dimension; they are then stacked together to combine their respective features. At this point, we obtain a feature layer of [C, 1, H + W], which undergoes further processing using convolution + normalization + activation functions to extract additional features;
3. The merged feature layer is subsequently separated back into two parallel stages: [C, 1, H] and [C, W]. They are obtained through transposition resulting in two separate layers: one with shape [C, H, 1] and another with shape [C, W];
4. The number of channels is adjusted by employing 1 × 1 convolution, followed by the application of the sigmoid function to obtain attention weights on the width and height dimensions. These weights are then multiplied with the original features, resulting in the output.

## 3. An Enhanced Approach for Fault Diagnosis in Capsule Networks

### 3.1. Development of a Dual-Branch Convolutional Capsule Neural Network for the Diagnosis of Rolling Bearing Faults Model

The capsule neural network employs a dynamic routing mechanism to replace the conventional max pooling technique in convolutional neural networks, thereby circumventing the issue of diminished recognition rates resulting from information loss in pooling layers. Algorithms based on this network model have been enhanced to more effectively explore fault information in rolling bearings.

Firstly, when converting to a two-dimensional feature image input, the lack of effective features poses a challenge. To enhance the computational efficiency of the network, we introduce the Inception V2 module to augment the width of the network model. This involves replacing a large 5 × 5 convolution kernel with two smaller 3 × 3 convolution kernels, thereby expanding the receptive field and reducing the parameter quantity while circumventing expression bottlenecks and enhancing linear expressive power. Additionally, incorporating a 1 × 1 convolution kernel in this structure aids in reducing computation without compromising output results.

Secondly, to minimize information loss during training, we incorporate the CA attention mechanism that comprehensively focuses on both the channel information and spatial positional information of the model. With regards to network depth, we adopt GoogleNet’s approach by integrating multiple identical Inception V2 modules (with unchanged inputs and outputs). We append seven layers of inception structures to amplify the network depth and refine the model accuracy.

Finally, designing this model as a dual-branch [20] one aims at separately extracting feature information for different types of rolling bearing faults and damage diameters through two branches. By fully leveraging fault information and mitigating challenges in feature extraction posed by single branches alone, it enhances the generalization ability of the model.

**Figure 5:**
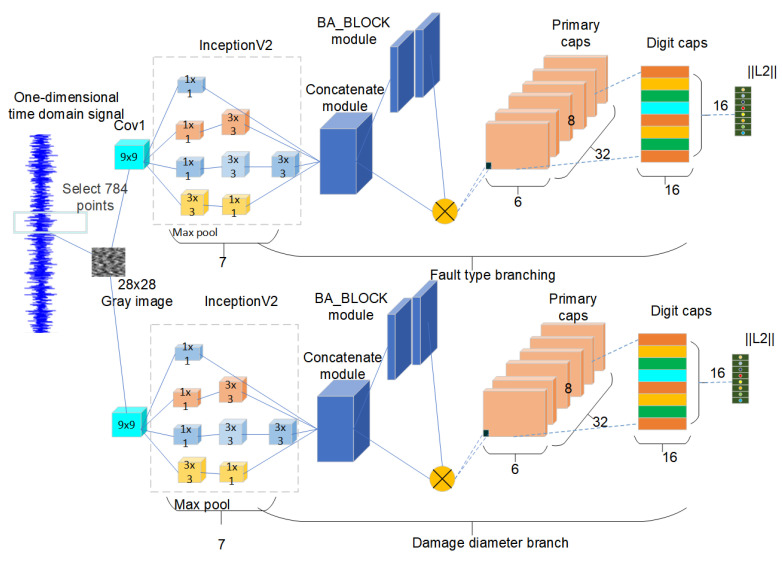
The model of the dual-branch convolutional capsule neural network rolling bearing.

The enhanced version of the capsule neural network is illustrated in Figure 5.

### 3.2. Process for Diagnosing Faults in Rolling Bearings

**Figure 6:**
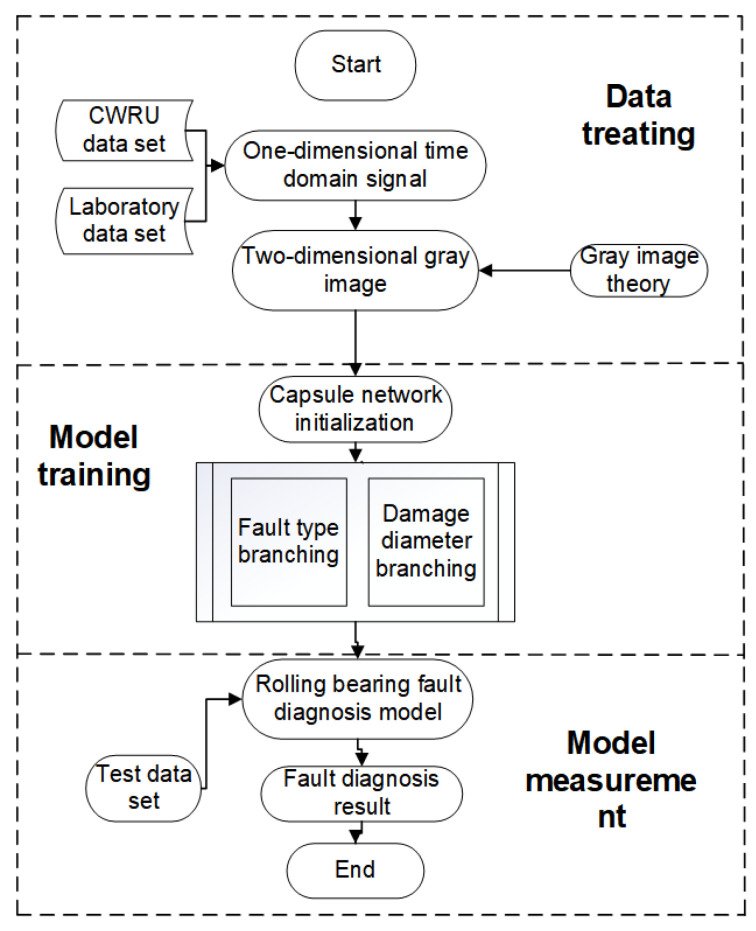
Fault diagnosis flow chart.

The fault diagnosis flowchart of the proposed dual-branch convolutional capsule neural network is illustrated in Figure 6. The specific steps are outlined as follows:

Step 1: The establishment of the original dataset is crucial, encompassing vibration signals from rolling bearings in various states, including different fault types and damage diameters. This comprehensive dataset will facilitate the verification of the model’s dual-label detection capability and diagnostic accuracy.

Step 2: Dataset preprocessing involves converting raw vibration signals from rolling bearings into grayscale images by assigning random numbers to consecutive data points. This step transforms the data into a two-dimensional image dataset that serves as input for the dual-branch convolutional capsule neural network.

Step 3: The training process of the diagnostic model includes dividing the two-dimensional image dataset into training and testing sets based on a specific ratio. The training set is utilized to train the diagnostic model effectively.

Step 4: Model testing entails conducting multiple experiments using test samples to validate the efficacy of this proposed model.

Step 5: To assess the generalization performance of our model, we employ a trained framework with publicly available datasets to train and validate data obtained during actual experimental processes. Continuous optimization of our model is performed based on real-world conditions, enabling subsequent diagnostics and decision-making tasks.

## 4. Experimental Design and Analysis of Results

### 4.1. Establishment of the CWRU Bearing Dataset

**Figure 7:**
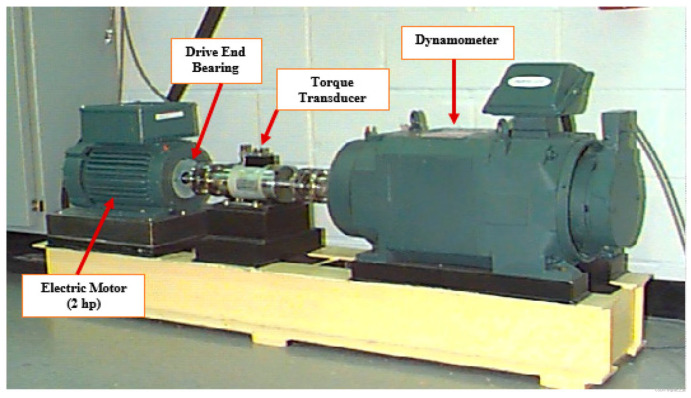
Test bench and rolling bearing.

The experimental data utilized in this study were obtained from the rolling bearing dataset provided by Case Western Reserve University (CWRU) [21]. The fault bearings examined on the test rig include the drive-end bearing (SKF6025) and the fan-end bearing (SKF6023). Vibration signals were acquired using a 16-channel data recorder with a sampling frequency of 12 kHz. Furthermore, for the drive-end bearing, additional data were recorded at a sampling frequency of 48 kHz, encompassing measurements taken at speeds of 1730 r/min, 1750 r/min, 1772 r/min, and 1797 r/min. Figure 7 illustrates the experimental setup.

The dataset comprises ten types of data, including one normal type and nine fault types. The nine fault types consist of inner race faults with a diameter of 0.18 mm, outer race faults with a diameter of 0.18 mm, rolling element faults with a diameter of 0.18 mm, as well as inner race, outer race, and rolling element faults with diameters of 0.36 mm and 0.54 mm, respectively.

One-hot encoding is utilized to categorize the classes using a ten-digit vector label representation. Based on the fault type (inner race, outer race, or rolling element) and damage diameter (0.18 mm, 0.36 mm, or 0.54 mm), each state is transformed from its original ten-dimensional feature vector into two four-dimensional feature vectors using binary one-hot encoding (where label 1 corresponds to the fault type while label 2 corresponds to the damage diameters). The fault types are classified as normal, inner race faults, outer race faults, or rolling element faults, whereas the damage diameters are categorized as normal (00 mm), 0.18 mm, 0.36 mm, or 0.54 mm. The dual-branch convolutional capsule neural network combines features extracted from both branches in four dimensions instead of ten dimensions in order to enhance its adaptive feature extraction capability for improved diagnostic performance in identifying different states based on Table 1, which presents the experimental samples and labels.

**Figure 8:**
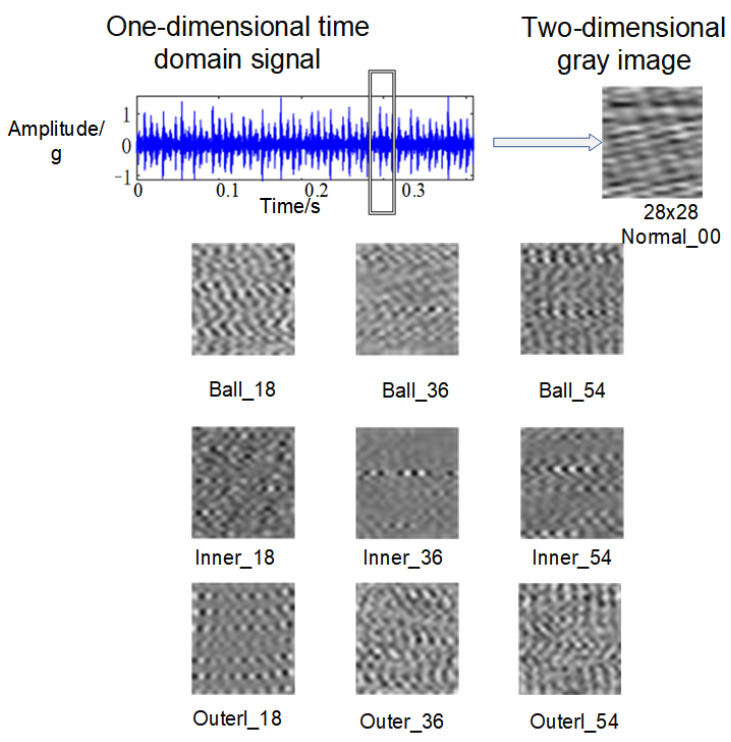
Grayscale sample set.

The term ‘ball_18’ in the aforementioned table denotes an inner race fault category characterized by a severity level of 0.18 mm. The chosen vibration signals originate from the drive-end bearing (SKF6025) and were acquired at a sampling frequency of 12 kHz. The dataset comprises ten distinct categories, wherein each category encompasses a contiguous set of 784 data points representing grayscale image samples. Every category undergoes independent random sampling for precisely 500 instances (once per rotational speed, where there are four kinds of rotational speed), thereby yielding a cumulative count of 2000 samples pertaining to this specific type. Overall, there exist twenty thousand samples distributed among the ten categories; furthermore, every individual sample is assigned two labels through the one-hot encoding methodology. The following Figure 8 represents an experimental grayscale sample image. For enhanced computational efficiency and streamlined extraction of crucial features, all samples are standardized to possess dimensions measuring at precisely 28 × 28. During the experiment, the training set and test set are randomly divided in a ratio of 4:1 using a fixed random seed input to ensure consistent results for each division.

**Figure 9:**
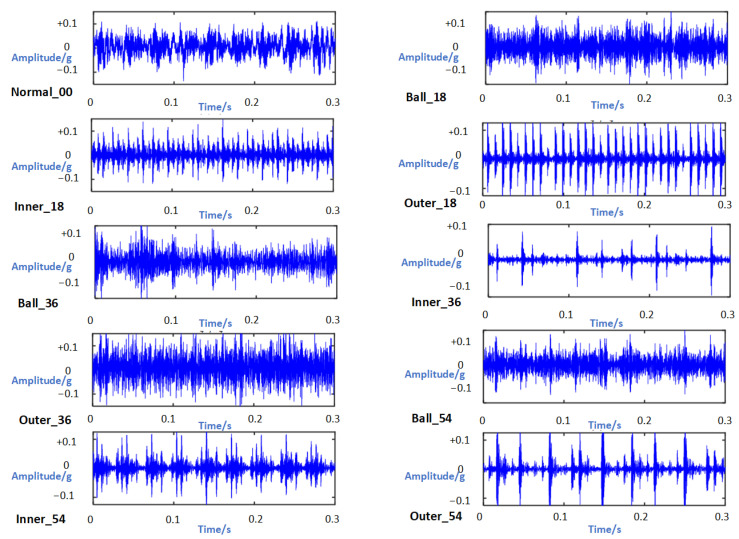
Time domain waveform.

Taking the 12 Hz operating condition of the drive end from the CWRU database as an example, we categorized the rolling bearing fault types and damage diameters into ten distinct categories. To ensure comprehensive capture of characteristic information in the bearing, a sampling point number of 1024 was set, and Figure 9 illustrates the resulting time domain waveform.

### 4.2. Model Parameter Configuration

The experimental setup for this study involved the utilization of the PyTorch deep learning framework on a Windows 10 operating system, with an Intel(R) Core(TM) i5-8300H CPU and NVIDIA GeForce GTX1050TI GPU. PyCharm was used as the editing tool, and the system had 16 GB of RAM. Python version 3.7.6 was employed for implementation purposes. During the model training process, optimization was performed using the Adam optimizer. The activation function for convolutional layers was set to ReLU, while capsule layers utilized the squash function as their activation mechanism. The hyperparameters were configured as follows: batch size = 16, maximum number of epochs = 50, learning rate = 0.001, and early stopping loss threshold = 0.0001. The primary capsule layer had a capsule vector dimension of 32, whereas the advanced capsule layer had a dimension of 256. Please refer to Table 2 for specific details regarding the parameter settings in our model.

The output size of a capsule unit in Table 2 is denoted as (6 × (8)), indicating that the feature layer has a width of six and each vector possesses eight dimensions. In the capsule layer, dynamic routing is employed to transform scalar features from previous convolutional outputs into feature vectors, which are subsequently operated on between capsule layers. Similarly, (16 × (10)) represents sixteen vectors with ten dimensions each, while (10 × (8)) signifies ten vectors with eight dimensions each. To leverage the strong fitting capability of capsule networks, dropout operations are incorporated in the capsule layer during training by randomly deactivating neurons at each iteration and freezing their weights. This strategy aids in reducing network complexity and mitigating overfitting.

### 4.3. Analysis of Experimental Results for CWRU Bearing Data

**Figure 10:**
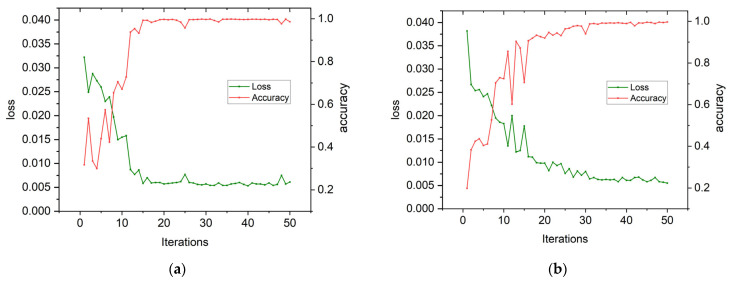
Branch fitting curve (CWRU). (**a**) Failure type branch fitting curve (CWRU). (**b**) Damage diameter branch fitting curve (CWRU).

Based on the parameters set in Section 4.2, Figure 10a,b depict the convergence curves of loss rate and accuracy for the test set. These curves demonstrate that while the fault type branch (Figure 10a) of rolling bearings converges after only 15 training sessions, it takes up to 30 sessions for the damage diameter branch to fully converge. The accuracy of the fault type branch steadily increases from 70.53% to 99.88%, with a decrease in its loss rate from 0.0322 to 0.0053. However, the fitting effect of the damage diameter branch (Figure 10b) is slower and reaches a maximum accuracy of only 99.72% at the last training session, with a final loss rate of 0.0055 after starting at an initial value of 0.0382. This confirms that our proposed dual-branch convolutional capsule neural network model is effective when utilizing specialized labels encoded with binary one-hot encoding input into both branches simultaneously.

**Figure 11:**
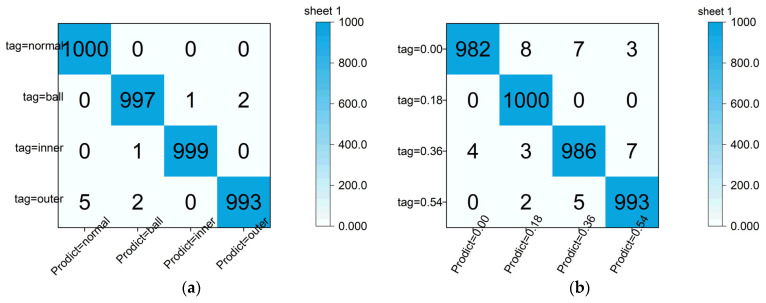
Confusion matrix (CWRU). (**a**) Failure type confusion matrix (CWRU). (**b**) Damage diameter confusion matrix (CWRU).

The confusion matrix of the test set in a certain experiment is illustrated in Figure 11a,b. The horizontal axis represents the predicted fault types (damage diameters) labels, while the vertical axis represents the actual fault types (damage diameters) labels. Each row indicates the number of samples correctly classified as a specific fault type or incorrectly classified as other fault types (damage diameters) within the test set. Each column denotes the number of test samples predicted for each fault type (damage diameter).

### 4.4. Experimental Evaluation of Noise Addition for Performance Testing

During operation, rolling bearings are susceptible to varying levels of external noise interference. To evaluate the proposed model’s anti-noise performance and generalization capability, Gaussian white noise with a specific signal-to-noise ratio (SNR) was introduced into the original samples. SNR serves as a crucial indicator for assessing the level of noise present in a signal, making its primary expression essential.

(2) $$SNR=10\times \mathrm{lg}\frac{P_{signal}}{P_{noise}}$$

In the above equation, *P_signal_* represents the power of noise and *P_noise_* represents the signal’s effective power.

**Figure 12:**
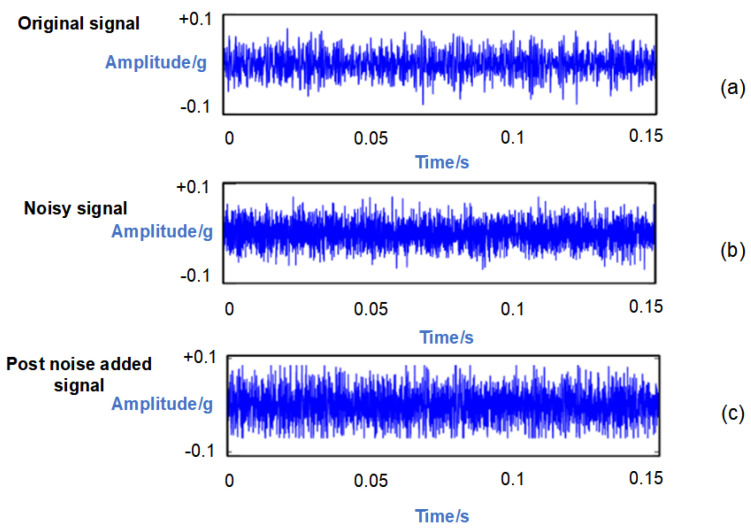
Time domain of different SNR states. (**a**) Original signal (the fluctuations are relatively moderate). (**b**) Noisy signal (the noise exhibits substantial fluctuations). (**c**) Post-noise-added signal (the time series is overwhelmed by noise).

Taking the outer ring fault signal at a rotational speed of 1750 r/min as an example, Gaussian white noise with a signal-to-noise ratio (SNR) of 3 dB is introduced to this signal. In order to visually demonstrate the process of noise addition, the original signal, noisy signal, and post-noise-added signal are individually plotted in the time domain waveform. As depicted in Figure 12, upon introducing noise to the outer ring fault signal, its temporal characteristics become submerged within the background noise, rendering it exceedingly challenging to discern the original waveform features.

In the noise addition experiment, various signal-to-noise ratios (−3 dB, 3 dB, 6 dB, and 9 dB) were employed to comprehensively assess the model’s robustness against diverse types of noise. Furthermore, the diagnostic accuracy of the model was separately evaluated under two conditions: dataset 1 (training set without noise, testing set with noise) and dataset 2 (both training and testing sets with noise). The recognition accuracy for the fault type and damage severity branches is presented in Table 3.

According to the data comparison presented in Table 3, it can be observed that dataset 2 generally exhibits a higher accuracy rate than dataset 1 by approximately 1–2% across different signal-to-noise ratios. This observation suggests that dataset 2 (training set with noise, testing set with noise) is more suitable for the proposed method. Specifically, under a −3 dB signal-to-noise ratio, dataset 2 achieves an accuracy rate of approximately 96%. Furthermore, when compared to the −3 dB signal-to-noise ratio, at a +3 dB signal-to-noise ratio, both the fault type branch and damage diameter branch show improvements in their respective accuracy rates by approximately 1.34% and 0.74%, respectively. In this study, multi-scale convolution was employed in the pooling layer to extract comprehensive information from fault data samples. Notably, when subjected to noise levels above 6 dB, the fault type branch demonstrates an accuracy rate exceeding 99%, while the damage diameter branch attains an accuracy rate surpassing 98%. These results indicate that our proposed method exhibits robustness against high levels of noise.

### 4.5. Comparative Validation of Diagnostic Efficacy across Diverse Algorithms

**Figure 13:**
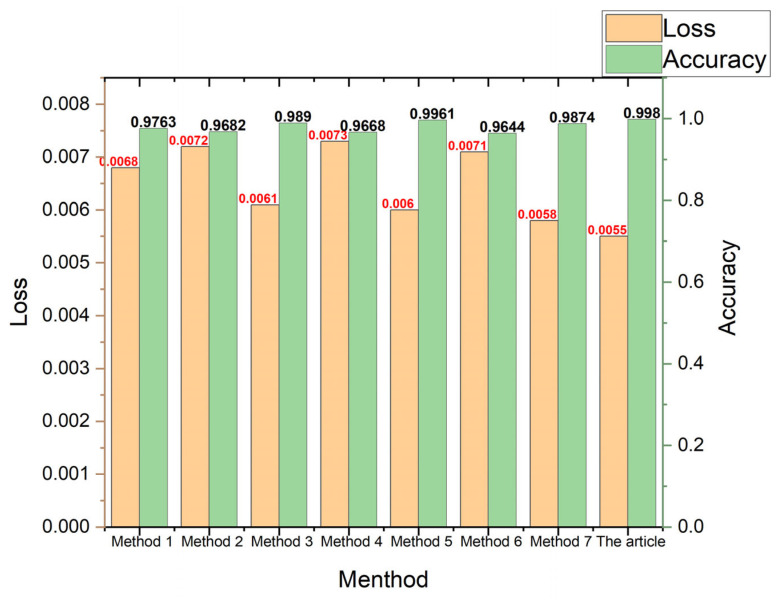
Identification accuracy and loss rate of each method.

The proposed model in this paper is validated for its superior diagnostic performance and compared with traditional single-label methods using binary one-hot encoding (two labels) on the dataset. Various algorithms are employed to diagnose the CWRU dataset. The algorithm model proposed in this paper represents a significant advancement over both the CNN and CapsNet architectures. Therefore, several models based on these networks are selected for comparison to demonstrate the superiority of the double-branch convolutional capsule neural network model. As the method described in this paper adopts a dual-branch approach, both accuracy and loss rates are calculated by averaging the results from both branches. Figure 13 illustrates the comparison of recognition accuracy among different methodologies.

1. In reference [22], a single tag is utilized for data labeling. The vibration signals undergo a short-time Fourier transform, and the resulting time-frequency feature maps are inputted into a convolutional neural network (CNN) with adjusted parameters tailored for fault diagnosis. Data labeling remains consistent with a single tag. The result is method 1 in Figure 13;
2. In reference [23], data are annotated using a singular tag. One-dimensional signals are converted into grayscale images and integrated with a CNN for the purpose of rolling bearing fault diagnosis. The result is method 2 in Figure 13;
3. In reference [24], data are annotated using a single tag, followed by feature extraction through a two-dimensional convolutional layer. The extracted features are then fed into capsule layers for fault diagnosis, where both primary and digit capsule layers employ dynamic routing algorithms to transform the feature vectors. The result is method 3 in Figure 13;
4. In reference [25], a singular label is employed to annotate the data, while one-dimensional temporal signals are fed into capsule neural networks for feature extraction. The fault diagnosis task is accomplished by leveraging two convolutional layers within the capsule neural network. The result is method 4 in Figure 13;
5. In reference [26], data are annotated with a single tag, and we propose a TF-RCNN model based on the utilization of time–frequency regions. This model leverages multiple regions characterized by TFR features, while also incorporating an attention module to enhance the classification efficacy for different types through advanced classification strategies. The result is method 5 in Figure 13;
6. Reference [27] introduces a single tag for labeling and proposes a multi-ensemble approach for rolling bearing fault diagnosis based on deep autoencoders (DAE). Multiple DAEs are trained with different activation functions to extract type-specific features, which are then merged into a feature pool. The final result is determined through majority voting among the classifiers of each sample set. The result is method 6 in Figure 13;
7. In reference [28], data were annotated with a singular label, proposing an enhanced AlexNet model for the diagnosis of rolling bearings. The optimal pre-training was determined based on the classification diagnostic rate. A modified calculation model was selected to reduce the parameter count and mitigate overfitting. Superior classification results were achieved by incorporating mixed concepts using classifiers. The result is method 7 in Figure 13.

The parameters set in the aforementioned literature are consistent with those of the original text. Similarly, for the CWRU dataset (with experiments conducted at a speed of 1750 r/min), training was iterated 50 times and divided into a 7:3 dataset split. The diagnostic results are depicted in Figure 13. While previous studies [22,23,24,25,26,27,28] employed a single-label approach to identify the fault positions and damage levels of rolling bearings, this paper proposes a dual-label marking method that takes into account both the fault types and damage diameters of rolling bearings. It can be observed that the proposed method in this study achieves an improvement in accuracy by 2.17%, 2.98%, 0.9%, 3.12%, 0.19%, 3.36%, and 1.06%, respectively, compared to the other seven mentioned methods. The method mentioned in this article also has the lowest loss rate.

### 4.6. Revising the Model’s Performance Generalization Verification

**Figure 14:**
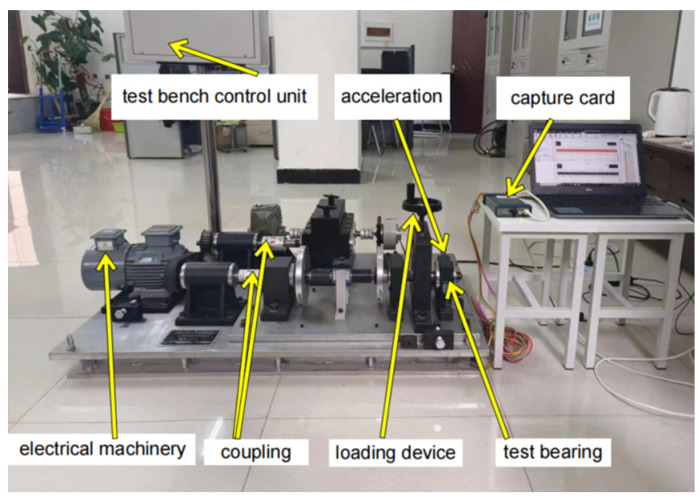
Experimental bench structure.

Due to the idealized nature of publicly available datasets, rolling bearings in real operating environments are exposed to various factors such as working conditions, temperature fluctuations, surrounding environmental noise, and electromagnetic interference during the data collection process. To further validate the generalization performance of this model on diverse datasets, we conducted data collection using our laboratory mechanical transmission simulation test rig. The experimental rig structure and sampling equipment are illustrated in Figure 14.

**Figure 15:**
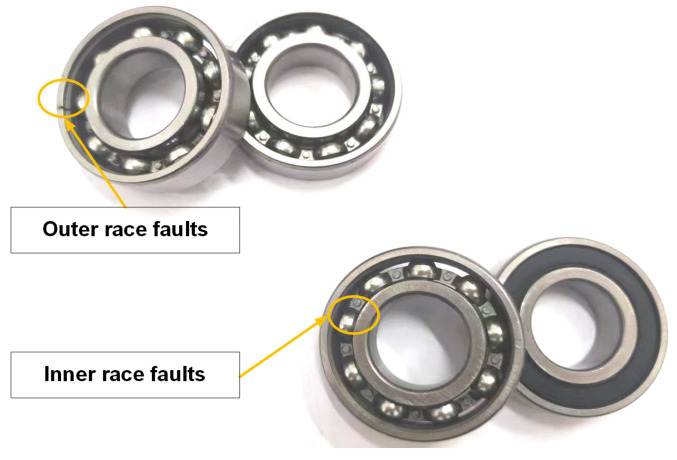
Fault bearing in the laboratory.

The experimental setup primarily comprises a motor, a drive shaft, a coupling, and a test bearing. Additionally, it incorporates data acquisition equipment and bidirectional channel acceleration vibration sensors for capturing vibration signals from diverse bearing types. The measured data are obtained from the outermost end of the base where an installed deep groove ball bearing with model number 6025 can be found. According to GB/T276-94 standards, this bearing has an inner diameter (d) of 25 mm, an outer diameter (D) of 52 mm, and a thickness (B) of 15 mm. The sampling frequency for this experiment is set at 12.8 kHz while maintaining the motor speed at 750 r/min. To ensure adequate collection time for each fault type, each sampling duration should not be less than 50 s. All fault bearings in this dataset have been artificially damaged through electric spark cutting techniques. Single-point faults with diameters measuring 0.18 mm, 0.36 mm, and 0.54 mm are intentionally placed on the inner race (inner), outer race (outer), and rolling elements (ball), respectively, whereas another category represents normal data without any discernible faults. The fault bearing in the laboratory is illustrated in Figure 15 below.

Data types: different fault types of rolling bearings. Training set (quantity): this is the quantity of samples for this type of training set. Test set (quantity): the number of samples for this type of training set. Under-loaded: the loading condition of this type of rolling bearing.

The term ‘ball_18’ in Table 4 represents a fault type of rolling element failure with a damage diameter of 0.18 mm. Similarly, ‘inner_36’ indicates a fault type of inner ring failure with a damage diameter of 0.36 mm, while ‘outer_54’ refers to a fault type of outer ring failure with the same damage diameter. The last column presents the data obtained under loaded and unloaded conditions, respectively.

**Figure 16:**
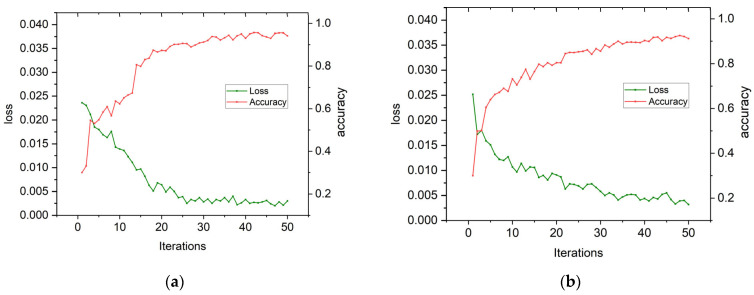
Branch fitting curve (self-made datasets). (**a**) Failure type branch fitting curve (self-made datasets). (**b**) Damage diameter branch fitting curve (self-made datasets).

The convergence curves of the model’s loss values and accuracy on our self-made dataset are illustrated in Figure 16. From these plots, it can be observed that the branch pertaining to fault types (Figure 16a) in rolling bearings essentially achieves convergence after 18 training iterations, while the branch concerning damage diameters requires up to 28 iterations for complete convergence. The accuracy of fault type classification steadily increases from 54.55% to 95.55%, accompanied by a decrease in loss rate from 0.0236 to 0.0020. On the other hand, the fitting effect of the damage diameters branch is not as rapid but continues to steadily improve, reaching a maximum accuracy of 92.88%. The corresponding loss rate decreases from 0.0252 to 0.0030.

The accuracy of the self-made dataset test set is only 94.25%, primarily due to the direct collection of vibration signals in the laboratory without prior noise reduction and filtering processes, resulting in their conversion into two-dimensional grayscale images. However, when compared to the accuracy achieved in Section 4.4’s experiment with a signal-to-noise ratio of −3 dB, there is an average decrease of approximately 1% observed in the self-made dataset’s accuracy. Consequently, this method exhibits commendable generalization performance across diverse datasets.

## 5. Conclusions

This article investigates the diagnosis method of rolling bearings using a dual-branch convolution CapsNet and presents the following conclusions:

1. The article proposes a novel diagnostic model for rolling bearings, which enables the identification of both fault type and damage diameters through a dual-branch structure. By effectively leveraging fault information to extract more intricate features, it significantly enhances the accuracy of diagnosis. Moreover, the adoption of a one-hot encoding binary labeling method reduces dimensionality and facilitates feature extraction in each branch while ensuring high precision;
2. The model was validated using the CWRU bearing dataset and a self-made dataset. The experimental results demonstrate that both branches of the model exhibit high accuracy, achieving an average accuracy of 99.8% for each branch on the CWRU dataset and an average accuracy of 94.25% for each branch on the self-made dataset. In comparison to four other fault diagnosis algorithm models in the existing literature, this model demonstrates a superior fault recognition rate and provides more comprehensive diagnostic information;
3. The model’s robustness against noise and superior generalization ability are demonstrated through experiments involving noise addition and evaluation of generalization performance.

The future will require further optimization of the methods outlined in this paper to enhance the stability and universality of network parameter selection under real-world operating conditions.

## Figures and Tables

**Table 1:** The experimental samples and labels.

Data Type	The Original Label	Label 1	Label 2
ball_18	0	[1,0,0,0]	[0,1,0,0]
ball_36	1	[1,0,0,0]	[0,0,1,0]
ball_54	2	[1,0,0,0]	[0,0,0,1]
inner_18	3	[0,1,0,0]	[0,1,0,0]
inner_36	4	[0,1,0,0]	[0,0,1,0]
inner_54	5	[0,1,0,0]	[0,0,0,1]
normal_00	6	[0,0,1,0]	[1,0,0,0]
outer_18	7	[0,0,0,1]	[0,1,0,0]
outer_36	8	[0,0,0,1]	[0,0,1,0]
outer_54	9	[0,0,0,1]	[0,0,0,1]

**Table 2:** The structural parameter design.

Number of Storeys	The Name of the Structure	Structural Parameters	Number of Channels	The Size of the Output
	Input	(28, 28)	1	28 × 28
1	(The Inception module)			
	Branch 1	(1, 1, 1)	48	28 × 28
	Branch 2	(1, 1, 1)/(3, 3, 1)/(3, 3, 1)	48	28 × 28
	Branch 3	(1, 1, 1)/(3, 3, 1)	64	28 × 28
	Branch 4	(3, 3, 1)/(1, 1, 1)	64	28 × 28
2	CA attention mechanism			
3	Basic capsule layer	(9, 9, 2)	32	6 × (8)
4	Numeric capsule layer	(10, 16, 1)	256	16 × (10)
5	Fully connected capsule layer	(256/1024)		10 × (8)
6	Output layer	(1024/10)		

**Table 3:** Noise experimental model accuracy.

Amplitude Branching	−3 db		3 db		6 db		9 db	
	Failure Type	Damage Diameter	Failure Type	Damage Diameter	Failure Type	Damage Diameter	Failure Type	Damage Diameter
Dataset 1	94.65%	95.26%	96.53%	95.32%	97.53%	96.53%	97.68%	97.32%
Dataset 2	97.34%	96.33%	98.68%	97.07%	99.03%	97.61%	98.47%	98.55%

**Table 4:** Laboratory homemade datasets and their construction.

Data Types	Training Set (Quantity)	Test Set (Quantity)	Loading Condition
ball_18	1600	400	Under-loaded
ball_36	1600	400	Under-loaded
ball_54	1600	400	Under-loaded
inner_18	1600	400	Under-loaded
inner_36	1600	400	Under-loaded
inner_54	1600	400	Under-loaded
normal_00	1600	400	Under-loaded
outer_18	1600	400	Under-loaded
outer_36	1600	400	Under-loaded
outer_54	1600	400	Under-loaded

## Data Availability

The data that support the findings of this study are available from the corresponding authors upon reasonable request.

## References

[1] Liu Y., Liu B. (2022). Residual analysis and parameter estimation of uncertain differential equations. Fuzzy Optim. Decis. Mak..

[2] Ng Y.S., Srinivasan R. (2010). Multi-agent based collaborative fault detection and identification in chemical processes. Eng. Appl. Artif. Intell..

[3] Skowronek K., Barszcz T., Antoni J., Zimroz R., Wyłomańska A. (2023). Assessment of background noise properties in time and time–frequency domains in the context of vibration-based local damage detection in real environment. Mech. Syst. Signal Process..

[4] Żuławiński W., Antoni J., Zimroz R., Wyłomańska A. (2024). Applications of robust statistics for cyclostationarity detection in non-Gaussian signals for local damage detection in bearings. Mech. Syst. Signal Process..

[5] Mauricio A., Smith W.A., Randall R.B., Antoni J., Gryllias K. (2020). Improved Envelope Spectrum via Feature Optimisation-gram (IESFOgram): A novel tool for rolling element bearing diagnostics under non-stationary operating conditions. Mech. Syst. Signal Process..

[6] Peng H.-M., Wang X.-K., Wang T.-L., Liu Y.-H., Wang J.-Q. (2021). Extended failure mode and effect analysis approach based on hesitant fuzzy linguistic Z-numbers for risk prioritisation of nuclear power equipment failures. J. Intell. Fuzzy Syst..

[7] François-Lavet V., Henderson P., Islam R., Bellemare M.G., Pineau J. (2018). An Introduction to Deep Reinforcement Learning. Found. Trends Mach. Learn..

[8] Guo P., Liu Z., Lu H., Wang Z. (2021). Hyperspectral Image Classification Based on Stacked Contractive Autoencoder Combined with Adaptive Spectral-Spatial Information. IEEE Access.

[9] Pouryahya M., Oh J.H., Mathews J.C., Belkhatir Z., Moosmüller C., Deasy J.O., Tannenbaum A.R. (2022). Pan-Cancer Prediction of Cell-Line Drug Sensitivity Using Network-Based Methods. Int. J. Mol. Sci..

[10] Xue Y., Wang Y., Liang J. (2022). A self-adaptive gradient descent search algorithm for fully-connected neural networks. Neurocomputing.

[11] Yin C., Wang Y., Ma G., Wang Y., Sun Y., He Y. (2022). Weak fault feature extraction of rolling bearings based on improved ensemble noise-reconstructed EMD and adaptive threshold denoising. Mech. Syst. Signal Process..

[12] Li Y., Xu M., Wei Y., Huang W. (2015). Bearing fault diagnosis based on adaptive mutiscale fuzzy entropy and support vector machine. J. Vibroengineering.

[13] Lu W., Mao H., Lin F., Chen Z., Fu H., Xu Y. (2022). Recognition of rolling bearing running state based on genetic algorithm and convolutional neural network. Adv. Mech. Eng..

[14] Ameer I., Bolucu N., Sidorov G., Can B. (2023). Emotion Classification in Texts over Graph Neural Networks: Semantic Representation is Better Than Syntactic. IEEE Access.

[15] Guo L., Li N., Jia F., Lei Y., Lin J. (2017). A recurrent neural network based health indicator for remaining useful life prediction of bearings. Neurocomputing.

[16] Luo Y., Jiang J., Zhu J., Huang Q., Li W., Wang Y., Gao Y. (2022). A Caps-Ubi Model for Protein Ubiquitination Site Prediction. Front. Plant Sci..

[17] Zhou Y., Jin L., Ma G., Xu X. (2022). Quaternion Capsule Neural Network with Region Attention for Facial Expression Recognition in Color Images. IEEE Trans. Emerg. Top. Comput. Intell..

[18] Yang B., Bao W., Wang J. (2022). Active disease-related compound identification based on capsule network. Brief. Bioinform..

[19] Zhang X., Sun Y., Wang Y., Li Z., Li N., Su J. (2019). A novel effective and efficient capsule network via bottleneck residual block and automated gradual pruning. Comput. Electr. Eng..

[20] Li Z., Lu C., Wang X., Ban S. (2022). A two-branch convolutional neural network fault diagnosis method considering rolling bearing fault location and damage degree. Sci. Technol. Eng..

[21] Min Q., He J.-J., Yu P., Fu Y. (2023). Incremental Fault Diagnosis Method Based on Metric Feature Distillation and Improved Sample Memory. IEEE Access.

[22] Li G., Deng C., Wu J., Chen Z., Xu X. (2020). Rolling Bearing Fault Diagnosis Based on Wavelet Packet Transform and Convolutional Neural Network. Appl. Sci..

[23] Wen L., Li X., Gao L., Zhang Y. (2018). A New Convolutional Neural Network-Based Data-Driven Fault Diagnosis Method. IEEE Trans. Ind. Electron..

[24] Sabour S., Frosst N., Hinton G.E. Dynamic routing between capsules. Proceedings of the 31st International Conference on Neural Information Processing Systems.

[25] Jiang G.-J., Li D.-Z., Feng K., Li Y., Zheng J., Ni Q., Li H. (2023). Rolling Bearing Fault Diagnosis Based On Convolutional Capsule Network. J. Dyn. Monit. Diagn..

[26] Huo C., Jiang Q., Shen Y., Qian C., Zhang Q. (2022). New transfer learning fault diagnosis method of rolling bearing based on ADC-CNN and LATL under variable conditions. Measurement.

[27] Kong X., Mao G., Wang Q., Ma H., Yang W. (2020). A multi-ensemble method based on deep auto-encoders for fault diagnosis of rolling bearings. Measurement.

[28] Mohiuddin M., Islam S., Islam S., Miah S., Niu M.-B. (2023). Intelligent Fault Diagnosis of Rolling Element Bearings Based on Modified AlexNet. Sensors.

